# Mechanical Behavior of Ultrahigh-Performance Concrete Tunnel Lining Segments

**DOI:** 10.3390/ma14092378

**Published:** 2021-05-03

**Authors:** Safeer Abbas, Moncef L. Nehdi

**Affiliations:** 1 Civil Engineering Department, University of Engineering and Technology, Lahore 54890, Pakistan; safeer.abbas@uet.edu.pk; 2 Department of Civil and Environmental Engineering, Western University, London, ON N6A 5B9, Canada

**Keywords:** ultrahigh-performance concrete, tunnel lining segment, durability, steel fibers, flexural load, thrust load

## Abstract

Ultrahigh-performance concrete (UHPC) is a novel material demonstrating superior mechanical, durability and sustainability performance. However, its implementation in massive structures is hampered by its high initial cost and the lack of stakeholders’ confidence, especially in developing countries. Therefore, the present study explores, for the first time, a novel application of UHPC, incorporating hybrid steel fibers in precast tunnel lining segments. Reduced scale curved tunnel lining segments were cast using UHPC incorporating hybrid 8 mm and 16 mm steel fibers at dosages of 1%, 2% and 3% by mixture volume. Flexural and thrust load tests were conducted to investigate the mechanical behavior of UHPC tunnel lining segments thus produced. It was observed that the flow of UHPC mixtures decreased due to steel fibers addition, yet steel fibers increased the mechanical and durability properties. Flexural tests on lining segments showed that both the strain hardening (multiple cracking) and strain softening (post-peak behavior) phases were enhanced due to hybrid addition of steel fibers in comparison with the control segments without fibers. Specimens incorporating 3% of hybrid steel fibers achieved 57% increase in ultimate load carrying capacity and exhibited multiple cracking patterns compared to that of identical UHPC segments with 1% fibers. Moreover, segments without fibers incurred excessive cracking and spalling of concrete at the base under the thrust load test. However, more stable behavior was observed for segments incorporating steel fibers under the thrust load, indicating its capability to resist typical thrust loads during tunnel lining field installation. This study highlights the potential use of UHPC with hybrid steel fibers for improved structural behavior. Moreover, the use of UHPC allows producing structural members with reduced cross-sectional dimensions, leading to reduced overall structural weight and increased clear space.

## 1. Introduction

The premature corrosion of steel rebar and associated degradation of concrete infra-structure has led to the development of new ultra-durable cement-based composite materials. New developments in supplementary cementitious materials, chemical admixtures, steel fibers and related technologies stimulated the introduction of the so-called ultrahigh performance concrete (UHPC). UHPC is distinguished by its very high compressive strength (at least 150 MPa (22 ksi)), non-brittle flexural behavior, high-impact and fatigue resistance, improved freezing and thawing resistance, high resistance to chemical attack and to chloride ions penetration [1,2,3]. UHPC requires specific mixture design, mixing sequence and mixing methods to ensure strong micro- and macro-internal structure along with very dense particle packing, leading to almost zero porosity [1,2,3,4]. This can be achieved through carefully selecting UHPC constituents and their particle size gradation, reducing the maximum size of aggregates, using low w/c, pozzolanic materials and adding steel microfibers to achieve ductile behavior [1,4,5,6,7]. Fiber distribution and its orientation in UHPC play a vital role in achieving the desired properties [8]. Steam or thermal curing of UHPC specimens also significantly influence the mechanical and durability properties [9]. In addition, curing under pressure can further densify the internal structure and improve the overall properties significantly. For instance, UHPC specimens subjected to 400 °C temperature and 50 MPa pressure can exhibit an increase in compressive strength up to 650 MPa [4]. The innovative UHPC material has a broad range of potential applications in constructing diverse structures and upgrading and rehabilitating existing ones [10,11,12,13,14,15,16,17,18,19]. The major disadvantage of UHPC is its high cement content leading to increased CO_2_ emissions and associated climate change. This can be addressed by incorporating supplementary cementitious materials (SCMs) or other filler materials as partial replacement for cement, thus reducing the environmental overburden [3,20,21]. Furthermore, fine recycled concrete materials can be used in producing UHPC, which minimizes the utilization of natural resources, attaining more sustainable construction [22].

Precast tunnel lining (PCTL) segments are an important component of tunnel infrastructure. PCTL segments have shown better performance in comparison with conventional in-situ concrete lining owing to their improved quality, and the ease of controlling and managing the installation processes through tunnel boring machines. Tunnel linings are subjected to various temporary and permanent loading conditions, for instance at their initial stage of casting. Moreover, additional stresses are induced in de-moulding and stacking of the segments, and during the installation of segments through tunnel boring machines (TBM), whereby thrust forces are exerted on the lining segments. Finally, after installation of the segments, loads imposed by the surrounding ground induce further bending and shear loads [23,24,25]. The interaction of a concrete member with its surrounding soil depicts a complex behavior, which needs to be examined at the initial stage for long-term improved performance of the structure [26]. Furthermore, stress transfer mechanisms between the longitudinal joints and segmental joints in precast linings are important factors that affect the stability of the overall structural system. The structural behavior of segmental tunnel linings needs to be modeled accurately for considering all the expected loads and surrounding conditions [27]. It should be noted that spalling of concrete during fire events in tunnels is a major concern, which needs considerable attention. It was reported that UHPC is more vulnerable to fire loading due to its dense microstructure [28]. However, the addition of polypropylene fibers in UHPC mixtures can limit such concrete spalling [28,29].

Various previous studies have explored the structural and durability performance of PCTL segments. For instance, Abbas et al. (2014) [23,30,31] studied the structural and durability performance of full-scale precast steel rebar reinforced concrete (RC) and steel fiber-reinforced concrete (SFRC) tunnel lining segments. It was observed that the RC segments showed higher ultimate load than that of the SFRC segments; however, an improved post-cracking behavior was observed for SFRC segments. Both types of tested segments satisfied the design load criteria [23,30]. Furthermore, it was observed that the SFRC segments achieved improved durability performance in comparison with the RC segments [31]. In another study, Sharghi et al. (2021) [32] reported the use of recycled SFRC in tunnel linings and studied the thrust load application imposed during installation of segments using finite element analysis. Qiu et al. (2021) [33] investigated the mechanical behavior, force transfer mechanism and failure criteria of segmental joints by performing various tests on prototype segments. Similarly, Liu et al. (2017) [34] reported the behavior of stagger joint by performing a ring test made up of actual tunnel segments.

Carbon fiber-reinforced plastics (CFRPs) were used for repairing tunnel segments and the structural performance under various loading regimes was assessed [35]. Nehdi et al. (2015) [36] investigated the performance of scaled tunnel lining segments made of UHPC incorporating various fiber length and dosages. It was reported that the segments with shorter fiber exhibited multiple micro-cracking and higher ultimate load compared to that of the segments incorporating long fibers. However, an improved post-peak behavior was observed for segments with longer fibers, leading to more ductile failure [36]. Similarly, Ni et al. (2018) [37] studied the flexural performance of tunnel lining segments made with UHPC incorporating 18 mm long steel fibers.

The use of fibers in precast tunnel linings produced remarkable structural and durability performance in terms of cracking behavior, strain hardening and post-peak behavior. The hybridization of steel and polypropylene fibers in UHPC mixtures attained significant improvement in structural and durability properties owing to their better cohesion with the matrix [38,39]. Steel fibers normally improve the mechanical and physical properties of the mixture, while polypropylene fibers can enhance durability performance through their chemically inert behavior [38,39]. Various studies in the past have been conducted on the hybridization of short and long fibers and concluded that it has beneficial effects in UHPC mixtures [40,41,42].

Normally, UHPC for large-scale production requires curing at elevated temperature (e.g., 90 °C) for 48 h. Thus, using UHPC in the precast industry requires special arrangements to alter the conventional curing regime. The uniqueness of this study lies in manufacturing the UHPC tunnel lining segments using the same curing regime already available in industrial precast plants. However, sustainable UHPC still achieved high early-age strength; therefore, its utilization in mega-scale production of tunnel lining segments can accelerate construction schedules, ultimately saving time and cost. Using UHPC in manufacturing PCTL segments further restricts the penetration of groundwater and harmful chemicals owing to the very low porosity of UHPC. The high strength properties of UHPC incorporating fibers provides a viable option for using slender sections of tunnel lining segments, hence reducing the overall cost of the project. Moreover, previous studies have concluded that while steel fibers can partially replace conventional reinforcing steel rebar cages in tunnel lining; this complete replacement demands a material having high structural strength properties. This issue can be solved by utilizing UHPC incorporating fibers in PCTL segments, which further adds novelty to this study. This study utilizes hybrid steel fibers in manufacturing UHPC tunnel lining segments and eliminates the costly premature corrosion deterioration of conventional RC segments.

Based on extensive survey of the pertinent literature, it can be argued that the performance of UHPC tunnel lining segments with hybrid short and long steel fibers can have synergistic effects leading to improved overall performance. However, this aspect is yet to be explored in the open literature. The original contribution of this study is to investigate the structural behavior (flexural and thrust load action) of UHPC tunnel lining segments made with hybrid steel fiber addition (8 mm and 16 mm) at various dosage levels (1%, 2% and 3% by mixture volume). This work was envisioned to enhance the strain hardening and strain softening characteristics via synergizing the individual capabilities of shorter and longer fibers in tunnel linings. Moreover, this study can motivate stakeholders to instigate the construction of mega-scale UHPC tunnel structures with superior mechanical performance and durability.

## 2. Materials and Methods

### 2.1. Materials and Specimen Preparation

**Figure 1:**
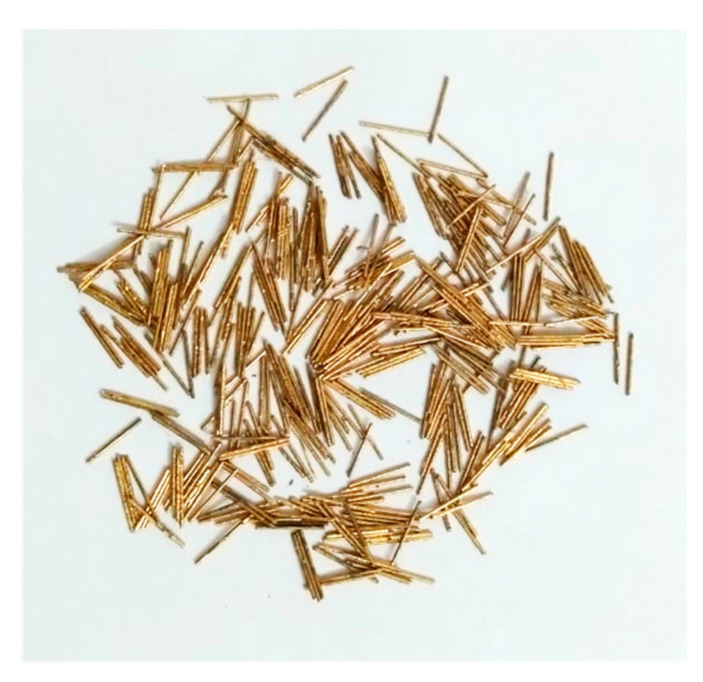
Steel fibers used for casting UHPC specimens.

Table 1 reports the chemical properties of used ordinary portland cement, silica fume, quartz powder and quartz sand. A polycarboxylate superplasticizer was used to control workability. Table 2 shows the mixture design of UHPC. Hybrid steel fibers having 8 mm and 16 mm in length were used at different dosage levels (1%, 2% and 3% by mixture volume). The hybrid fiber mixture consisted of 50% 8-mm and 50% 16-mm fibers. The used steel fibers were copper coated and straight (Figure 1). The diameter of the fiber was 0.25 mm and achieved uniaxial tensile strength greater than 2800 MPa.

**Figure 2:**
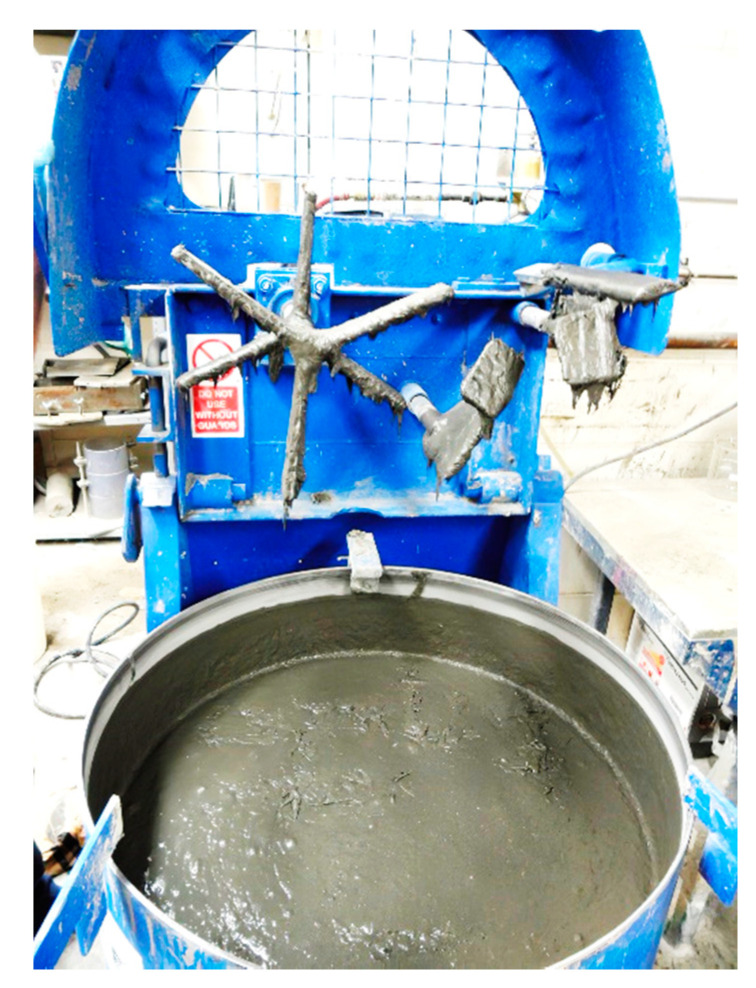
Shear mixer for mixing of UHPC.

Initially, dry mixing of all the ingredients (except the steel fibers) was carried out using a shear pan mixer (Figure 2). Afterwards, water premixed with the superplasticizer was gradually added into the mixture. Fibers were added into the mixture in the final stage of mixing.

**Figure 3:**
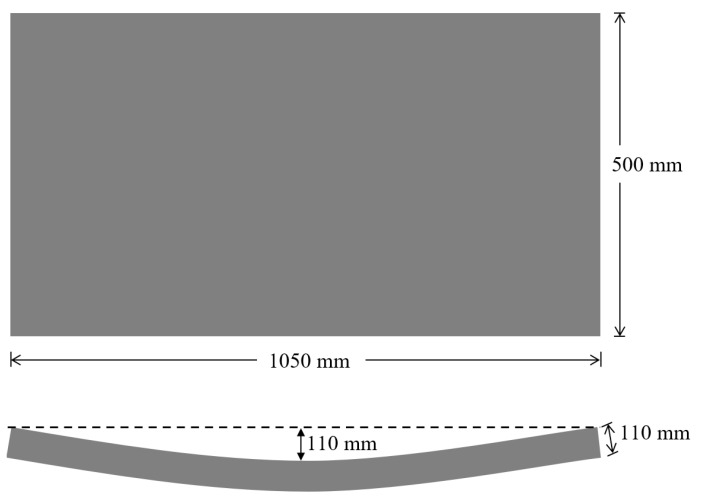
Dimensions of tested UHPC tunnel lining segments.

The mixing was ended when a uniform distribution of steel fibers was achieved without any fiber clumping. After mixing, tunnel segments having 1050 mm in length and 500 mm in width were cast on a vibratory table to achieve adequate consolidation. The crown height and thickness of the cast specimens was 110 mm (Figure 3). The selected size of the segments was a one-third-reduced replicate of the full-scale segments used in a real tunnel project in Toronto, Canada.

The segment mold was placed on a table in such a way that its extrados face points upward (convex face up). The UHPC mixture was then poured into the segment at its midspan, allowing the mixture to flow on both sides. Similar concrete pouring direction was adopted in an industrial precast plant for the manufacturing of full-scale lining segments. After pouring the UHPC mixture into segment molds, they were transported to an environmental chamber at 45 ± 2 °C and relative humidity (RH) greater than 95%. This curing regime was adopted to mimic the curing regime adopted in industrial scale production by precast plants. After 24 h, the segments were removed from their respective molds and transferred to a moist curing room at 20 °C and RH > 95% for 28 days until testing. Two segments for each studied variable were cast to capture statistical variability. Cylindrical specimens were also made and cured in a similar fashion for measuring the mechanical and durability properties of the various UHPC mixtures.

### 2.2. Testing Procedure and Methodology

#### 2.2.1. Material Properties of UHPC Mixtures

The flow characteristics of the freshly mixed UHPC incorporating various proportions of hybrid steel fibers were measured using a mini slump cone test. The diameter at four various locations was recorded and the average value was reported. The compressive and splitting tensile strengths of UHPC cylindrical specimens incorporating hybrid steel fibers at various dosages (1%, 2% and 3%) were determined as per ASTM C39 [43] and ASTM C496 [44], respectively. The loading rate for compression and tensile testing was 0.95 MPa/sec and 0.025 mm/min, respectively. The volume of permeable voids (VPV) and water sorptivity were also measured on 50-mm-thick specimens as per ASTM C642 [45] and ASTM C1585 [46], respectively. To access the chloride ions penetration into UHPC specimens, rapid chloride ions permeability testing (RCPT) was conducted in accordance with ASTM C1202 [47] guidelines. The total number of tested specimens and their coefficient of variance (COV) for the different tests performed are given in Table 3.

#### 2.2.2. Flexure Test on UHPC Segments

**Figure 4:**
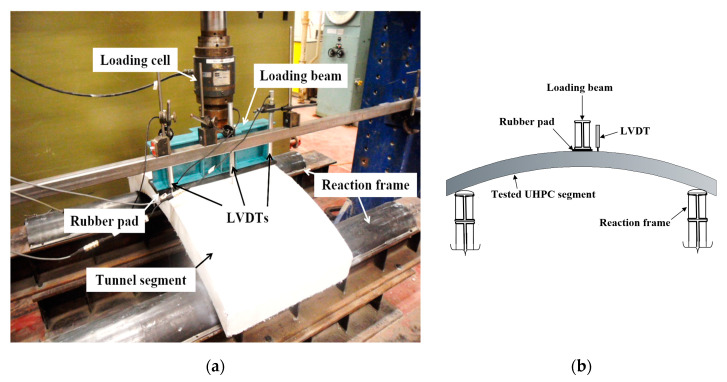
Flexural test setup of UHPC lining segment: (**a**) Experimental test setup; (**b**) Schematic test setup (side view).

The three-point load test was performed on UHPC PCTL segments to investigate its bending behavior. Figure 4 shows the test setup for flexure test on tunnel lining segment. UHPC tunnel lining segments were placed on a cylindrical support reaction frame with their convex face upward (extrados face upward). The reaction frame was continuous on both sides along the width of the segment and connected to the ground floor through bolts. A loading frame was placed at the center of the segment. A rubber pad was placed in between the loading frame and segment for ensuring uniform distribution of the load by adjusting the minor uneven surfaces at the extrados face of the segment. An incremental load at a rate of 0.50 mm/min was applied using loading stroke. Three linear variable displacement transducers (LVDTs) were installed at the mid span of the segments for measuring the vertical deflections (displacement) (Figure 4). Data was continuously recorded through an automated data-acquisition system. All tested segments were painted with a white color for easy monitoring of the crack development during the progression of load. The crack width was recorded using a graduated crack width ruler.

#### 2.2.3. Edge Point Test on UHPC Segments

**Figure 5:**
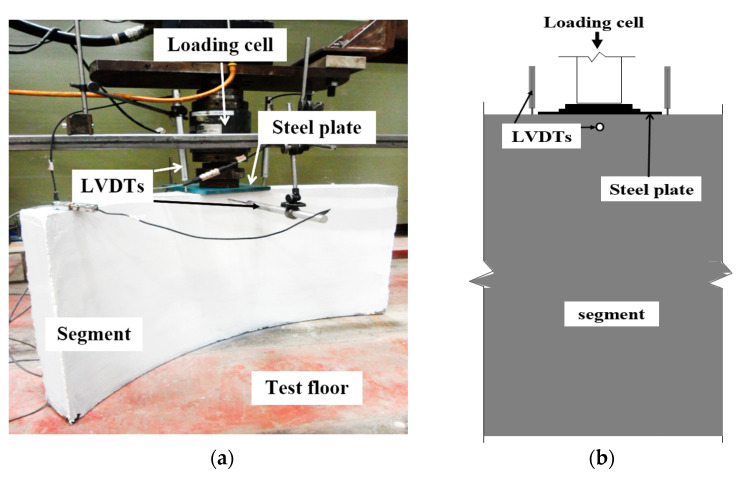
Thrust load test setup of lining segment: (**a**) Experimental test setup; (**b**) Schematic test setup (front view).

UHPC tunnel lining segments were tested for edge point load to investigate their behavior under thrust loads imposed through tunnel boring machine during in-situ installation of the precast segments. Figure 5 depicts the experimental setup for replicating the thrust load on UHPC segments. PCTL segments were vertically placed on the reaction floor. The load was applied at a rate of 2 mm/min via a square steel plate placed at the center of the segment. Each side displacement of the segments was monitored through LVDTs placed at the segment intrados and extrados surfaces. Moreover, LVDTs were installed near the loading arrangement for measuring the vertical deflections. Cracks were monitored upon the application of the load on the UHPC segments. A similar test setup (Figure 5) was used in previous studies and proved accurate and reliable [23,36].

## 3. Results

### 3.1. Material Characterization of UHPC

Table 4 illustrates the mechanical properties of the tested UHPC mixtures with various dosages of hybrid steel fibers. The flow of the fresh control mixture without fiber addition was 810 mm. It was observed that the flow decreased when steel fibers were incorporated in the mixture. For example, the flow was 792 mm and 752 mm for UHPC mixtures incorporating 1% and 3% of steel fibers by mixture volume. It is well established that steel fibers hinder the mixture velocity under free flowing. Further, the hybrid short-long steel fibers can limit the rotation of individual fibers, leading to decreased flow. Similar observations were reported in previous studies [2,48]. The compressive strength of the UHPC mixture without fibers was 148 MPa at 28 days. An increase in compressive strength was observed for specimens incorporating hybrid steel fibers. For instance, an increase in compressive strength at 28 days of approximately 8% was measured for specimens incorporating 2% of hybrid steel fibers. The hybridization of shorter and longer fibers restricted the initiation and propagation of internal micro-cracks and the associated internal material damage. Further, steel fibers in concrete specimens limit the axial and lateral deformations, thus leading to enhanced load carrying capacity [49,50]. It was observed that UHPC specimens without steel fibers failed in an abrupt manner with a loud sound and concrete detachment was observed. However, the UHPC specimens incorporating steel fibers remained intact without any breakage into pieces. No significant effect of fibers on the modulus of elasticity of the tested UHPC specimens was observed (Table 4).

Table 4 also reports the splitting tensile strength results of UHPC specimens. It can be observed that the incorporation of hybrid fibers in UHPC significantly enhanced the splitting tensile strength. For example, the splitting tensile strength of the fibreless control mixture was 9.50 MPa, while the mixture with 3% hybrid steel fibers reached a tensile strength of 21.52 MPa, which is a 226% of the value measured for the control mixture. It was further observed that the specimen incorporating the shorter and longer fibers effectively bridged the micro-cracks and did not allow complete splitting of the tested specimens into two parts.

Table 5 shows the durability properties of the tested UHPC mixtures. The control fibreless specimens had a volume of permeable voids (VPV) of around 3.5%. A decrease in VPV was observed for specimens incorporating hybrid steel fibers. The steel fibers played a significant role in reducing the capillary pore size by altering its continuity. Similar observations can be made for the sorptivity results (Table 5). The secondary sorptivity was 0.0430 and 0.0348 kg/m^2^/h^0.5^ for the UHPC specimens without fibers and with 2% of hybrid steel fibers, respectively. All the tested UHPC specimens achieved RCPT values of less than 100 coulombs, indicating negligible penetrability of chloride ions in accordance with ASTM C1202. Similar observations were reported in previous studies [2,51].

### 3.2. Flexural Behavior of UHPC Tunnel Lining Segments

All reported results of flexural behavior of UHPC segments were calculated as the average two specimens. A significant effect of steel fibers on the flexural capacity of UHPC segments can be observed. For instance, the control lining segment without fibers failed abruptly into two pieces at a load of around 18 kN without exhibiting any post-peak behavior. An increase in the ultimate load of approximately 53% was observed for the UHPC lining segments incorporating 1% of hybrid steel fibers compared to that of the reference UHPC segment without fibers.

**Figure 6:**
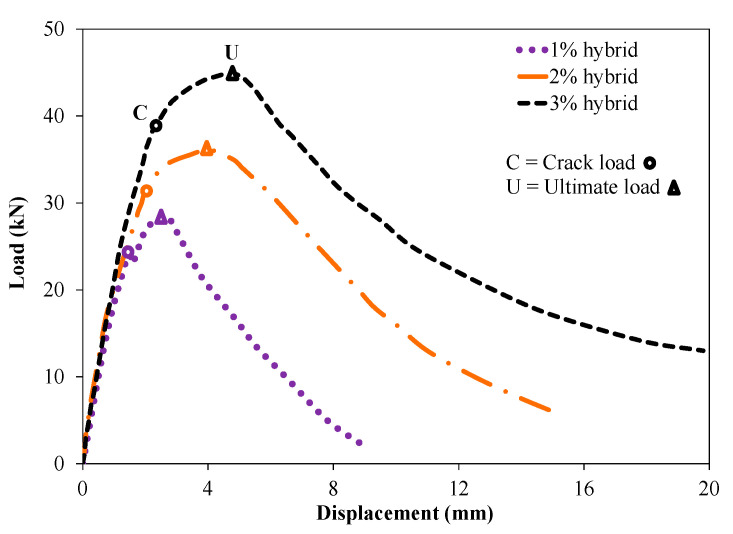
Load–displacement curve of UHPC lining segments under flexural loading.

Figure 6 depicts the load–displacement curve for UHPC lining segments incorporating various proportions of hybrid steel fibers. The displacement in Figure 6 displays the average values obtained from three LVDTs installed at the mid span of the segments. The load–displacement curve of UHPC lining segments incorporating various proportions of hybrid steel fibers can be divided into three zones: (i) a linear elastic portion from zero to the cracking point; (ii) a strain hardening zone from the onset of cracking to reaching the ultimate load and (iii) a strain softening region from the ultimate to the rupture point. For all the tested segments incorporating hybrid steel fibers, it was observed that the slope of the initial curves was linear and comparable. Due to the development of micro-cracks, the slope of the curves changed, which represents a reduction in the stiffness and start of the strain hardening zone. This zone was dependent on the number of fibers bridging the multiple cracks that have developed, until the pulling out of the fibers from the matrix. After reaching the ultimate load, a decrease in load was observed, and the strain softening phase started. In this zone, a continuously tickling sound was recorded associated with the rupture and pulling out of fibers from the cementitious matrix.

It should be noted that the observed behavior of the tested UHPC tunnel lining segments captures the effects of the incorporated hybrid steel fibers of 8 mm and 16 mm. The short fibers (8 mm) allow the development of multiple thin width cracking (instead of a single large crack) owing to its restricting capability on the growth of micro-cracks. On the other hand, longer 16 mm fibers have the capability to improve the post-peak behavior due to their larger embedment length, leading to more energy requirement for pulling out of fibers from the matrix. Therefore, it can be argued that the beneficial synergistic effects of hybrid shorter and longer fibers improved the structural efficiency of the overall system. Moreover, the development of additional numerous multiple cracks, associated with the strain hardening zone and post-peak behavior, was also dependent on the fiber dosage. It can be observed that the lining segments incorporating 3% of fibers (50%: 8 mm; and 50%: 16 mm) had a larger number of cracks and relatively higher extent of the strain hardening zone compared to that of identical segment with only 1% fibers (50%: 8 mm; and 50%: 16 mm). Similarly, higher fiber dosage (i.e., 3%) resulted in more gradual decrease in the load carrying capacity of the segments (descending branch) and exhibited larger deformation before failure compared to that of the steeper decrease in load for specimens made with lower fiber dosage (1%) (Figure 6).

Table 6 reports a summary of experimental results for the tested UHPC tunnel lining segments. Each result reported in Table 6 is the average value measured on two identical specimens. It can be observed that the segment incorporating 1% of hybrid steel fibers achieved cracking load and ultimate load of 25.5 kN and 28.5 kN, respectively. An increase in the cracking and ultimate loads was observed for segments made with higher dosage of steel fibers. For instance, UHPC segments incorporating 2% and 3% of hybrid steel fibers attained increases of around 24% and 55% in the crack load and ultimate load, respectively, compared to that of the identical segment with 1% of hybrid steel fibers. In the case of higher fiber dosage, a larger number of fibers were closely spaced, which better restricts the transformation of multiple microcracks into macrocracks. Therefore, owing to the delayed formation of a single macro-crack, an increase in the ultimate strain and ultimate load capacity was observed for segments incorporating higher dosages of hybrid fibers.

**Figure 7:**
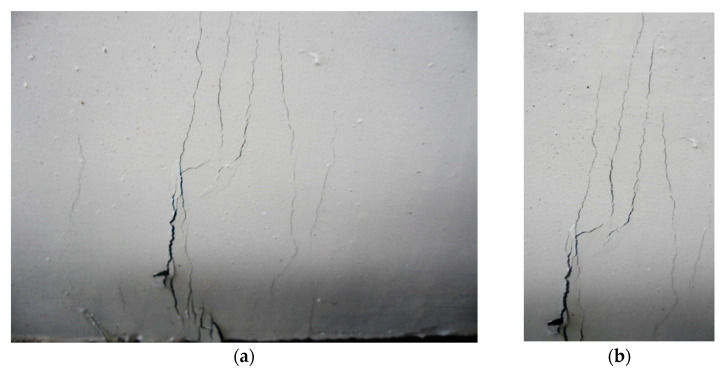
Typical cracking at the bottom of the UHPC segments. (**a**) Cracking at intrados face of segment, (**b**) Closer view of cracks.

**Figure 8:**
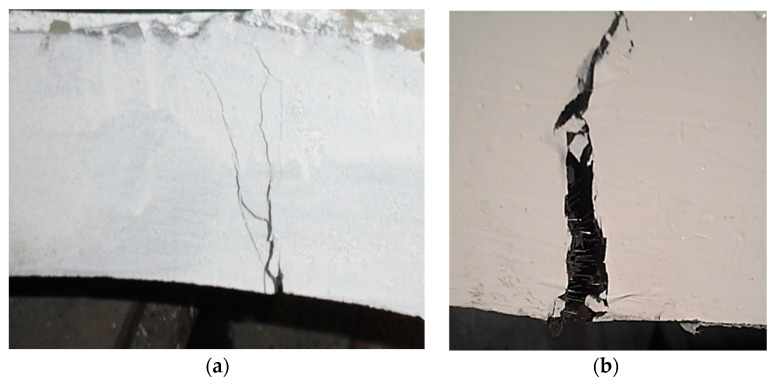
Cracking of UHPC segments along the thickness: (**a**) Cracks developed during testing; (**b**) Single macro crack leading to failure of the segment.

During the initial elastic portion of the tested UHPC lining segments, no cracks were detected. Firstly, discontinuous cracks appeared at the segment intrados face in the form of thin microcracks after exceeding the elastic zone (Figure 7). The crack spacing was around 60 mm for the tunnel lining segment incorporating 1% of hybrid steel fibers. The crack spacing was decreased for the segments incorporating higher dosage of fibers. Due to load increments, further tightly spaced microcracks developed, extended in both directions and perpendicular to the main tensile stress. Cracks also propagated towards the extrados faces along the thickness of the lining segment (Figure 8). A larger number of multiple microcracks were observed for segments incorporating higher dosages of steel fibers compared to that of an identical segment with a lower dosage of fibers before reaching the ultimate load. Fibers were pulled-out when the matrix ability to hold the fibers was exhausted. This resulted in stress concentrations so that further pulling out of fibers took place along with the development of new cracks and further widening of the previously developed cracks. Similar observations were reported in previous studies [36,52,53,54,55].

**Figure 9:**
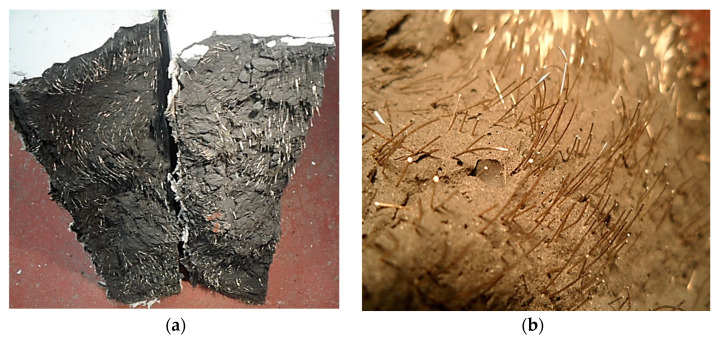
Failure of UHPC segments: (**a**) Segment broke into two pieces; (**b**) Fibers pulled out from the matrix.

In the strain softening zone of the tested UHPC tunnel lining segments, localized development of a single macrocrack (Figure 8) occurred due to increased stress concentration due to the pulling-out of highly stressed fibers. After failure of the segments into two pieces (Figure 9a), they were visually inspected and it was found that the pulling-out of fibers from the cementitious matrix was major compared to the limited occurrence of fiber fracture (Figure 9). It can be argued that the use of hybrid short and long fibers in UHPC tunnel lining segments can be beneficial for achieving more desirable strain hardening and stain softening zones, along with the development of multiple cracks under potentially severe service life loading conditions. Ni et al. (2018) [37] also studied the bending behavior of UHPC tunnel segments and reported an improved flexural behavior of UHPC segments compared to that of the RC segment [37]. However, hybrid fibers were not used in their study.

### 3.3. Thrust Behavior of UHPC Tunnel Lining Segment

The design of tunnel lining segments against thrust loading applied through the shoving forces exerted by tunnel boring machines during full-scale installation is generally the most critical load case, which can lead to severe damage during the installation process, thus compromising the structural strength of the lining and leading to wastage of segments. A comparable result (load versus deflection) was observed for segments incorporating steel fibers (1%, 2% and 3%). Therefore, to avoid overlap in the graph, results of UHPC segments with 2% hybrid steel fiber and the control segment without fibers were presented.

**Figure 10:**
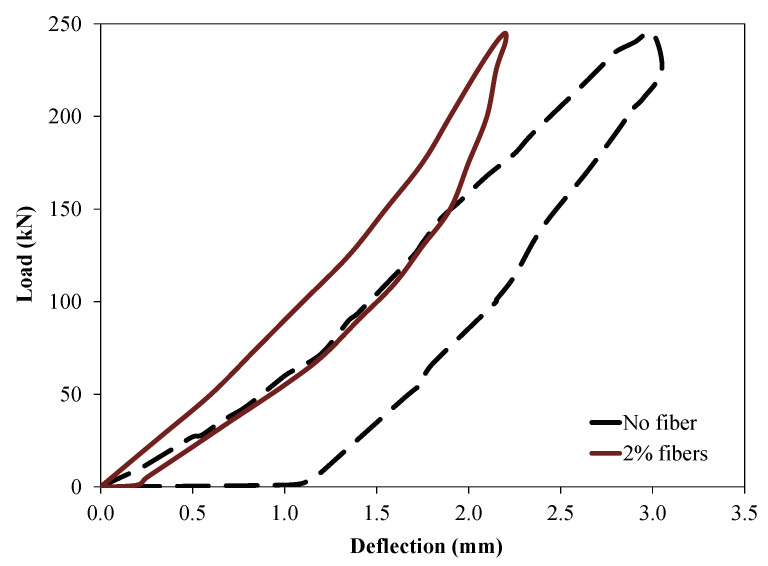
Thrust load test results on reduced scale PCTL segments made with UHPC.

Figure 10 depicts the behavior of UHPC under thrust loading action. The tested reduced scale tunnel lining segments were loaded to the maximum loading capacity of the used setup (250 kN). Higher initial stiffness was observed for UHPC segments incorporating 2% of hybrid steel fibers compared to that of the reference segment without fiber addition. It can be observed that the control UHPC segments without fibers incurred much higher vertical deflection compared to that of identical UHPC segments incorporating 2% of hybrid steel fibers. No cracks under the applied point load were observed for the UHPC segments incorporating 2% fibers. However, the segments without fibers suffered significant cracking under the point load at the base of the segment near to the test floor. Initially, minor cracks were observed at the bottom of the lining segments without fibers near the test floor at a load level of 82 kN. These cracks were further widened and splitting of concrete was evident at a load level of 223 kN for the segment without fibers. Similar observations were reported in previous study [36]. Meda et al. (2016) [56] conducted a study on full-scale lining segments under thrust loads. Various experimental test set-ups were investigated for simulating the actual stresses induced during the installation process of the lining segments through TBM. It was reported that the cracks were developed at the segment base, which was further confirmed through numerical analysis [56]. Similarly, Tiberti et al. (2015) [25] studied the local splitting behavior that may have been induced because of thrust action produced by TBM, and identified the positive impacts of fibers in restricting the splitting cracks under loading points.

## 4. Discussion

The flowability of the tested UHPC mixtures was affected by the addition of steel fibers. For instance, approximately 8% decrease in flow was observed for the mixture incorporating 3% of hybrid steel fibers compared to that of the control mixture without fibers. This is apparently because steel fibers alter the skeleton structure of the granular particles and increase friction of the matrix, thus causing hindrance against the free flow of fresh paste. It was observed that in comparison with long fibers, short fibers can lead to improved flow for the same fiber dosage in the mixture [2]. Further, hybridization of short and long fibers could strike an optimum balance of the desired mixture flow and mechanical performance. A relative increase in compressive strength was observed for the mixture incorporating hybrid steel fibers. For example, compressive strength of UHPC specimens incorporating 1% and 3% of hybrid steel fibers was 157 and 164 MPa respectively, compared to that of the control specimens’ compressive strength of 148 MPa. On the other hand, the splitting tensile strength of the tested UHPC mixture was significantly influenced by the addition of hybrid steel fibers. For instance, UHPC specimens with 1% and 3% of hybrid steel fibers exhibited splitting tensile strengths of around 15.8 and 21.5 MPa, respectively, compared to 9.5 MPa for the control specimens without steel fibers. It should be noted that hybrid fibers were added in UHPC primarily to improve the tensile properties of the mixture; however, improved compressive strength of UHPC owing to hybrid fiber incorporation was an additional benefit, leading to further improvement in structural performance. No fiber clusters were observed in the tested UHPC specimens, rather, a homogenous distribution of fibers was noted. Improved durability performance of tested UHPC mixture incorporating hybrid steel fibers was evident from the experimental testing. It was observed that the VPV for specimens incorporating 3% of steel fibers was 3.01% compared to 3.51% for control mixture without fibers. The RCPT of UHPC specimens showed coulomb values of 54 and 46 for UHPC specimens incorporating 2% and 3% of hybrid steel fibers, respectively, demonstrating its capability to restrict the ingress of aggressive species into the cementitious matrix.

It was observed that the mechanical performance of UHPC segments with hybrid steel fibers was significantly improved compared to that of the control identical segment without fibers. The control UHPC lining segment without fibers failed at around 18 kN without exhibiting strain hardening and strain softening zones. The addition of hybrid steel fibers improved the cracking and ultimate loads of the lining segments. For instance, the UHPC lining segment incorporating 1%, 2% and 3% of hybrid steel fibers achieved ultimate loads of around 28, 36 and 45 kN, respectively. Furthermore, it was noted that a higher dosage of hybrid steel fibers led to improving the strain hardening and strain softening phases of the tested tunnel lining segments. An improved cracking behavior of UHPC incorporating fibers was also observed, in agreement with previous study [37]. The tested segments in the present study only used hybrid steel fibers as the main reinforcement. The combined use of reinforcing rebar and steel fibers can further improve the mechanical behavior of UHPC segments. Meng et al. (2016) [55] conducted a study on full-scale tunnel segments reinforced with both steel rebar and steel fibers and concluded their beneficial effects in terms of initial cracking and ultimate strength [55]. For the flexural test performance of the tunnel lining segment, a simply supported test setup was used, in agreement with previous studies [56,57,58]. The test setup was carefully selected for targeting the critical case of flexure testing of segments. However, it should be noted that in a real scenario, the circumferential movement or deformations of individual lining segments are restricted by other surrounding segments, which may increase the ultimate capacity of the lining segment. Therefore, in future testing, other support conditions need to be investigated for simulating diverse and more realistic field support conditions. The behavior of the tested segments incorporating fibers under thrust loading action showed improved performance compared to that of the control segment without fibers. Similar findings were reported in previous studies [25,58].

Experimental results on the mechanical performance of UHPC tunnel lining segments incorporating hybrid steel fibers open the door for ample opportunities to innovate mega-scale tunnel lining segments with superior sustainability and service life performance advantages. PCTL segments produced with conventional concrete have limited load capacity, low ductility and restricted post-peak behavior. Often, in field exposure, the ingress of chloride ions initiates corrosion and compromises the longevity of tunnel systems. Conversely, PCTL made with UHPC are more resilient under installation and service loads, achieve superior ductile behavior and enormously restrict the ingress of chloride ions, thereby preventing corrosion problems. The much stronger UHPC incorporating hybrid fibers permits producing slender segments with reduced deadload, which enables further advantages in their transport from the precast plant to the job site and during field installation using tunnel boring machines.

## 5. Conclusions

This study examined the mechanical behavior of UHPC tunnel lining segments incorporating various proportions of hybrid 8 mm and 16 mm long steel fibers. The tested variable included the fiber dosage of 1%, 2% and 3% by volume mixture. For each tested dosage, 50% of 8 mm and 50% of 16 mm fibers was used. Based on the experimental results, the following conclusions can be drawn:

1. The flow of the control fresh ultrahigh-performance concrete (UHPC) mixture without fibers was 810 mm. The flow of UHPC mixtures decreased due to the incorporation of hybrid fibers as expected, but adequate consolidation was readily achievable. For instance, flowability was decreased to 773 mm for the mixture incorporating 2% of hybrid steel flow. This decreased flow was mainly attributed to restricting free flowing of the mixture due to the addition of fibers, which act as a barrier and increase the interparticle friction.
2. A relative increase in the compressive strength was observed owing to the addition of hybrid steel fibers in UHPC mixtures. For example, an increase in compressive strength of about 10% was observed for specimens incorporating 3% of steel fibers compared to that of the specimens without fibers. This increase in compressive strength can be ascribed to the resistance against lateral and axial deformation offered by steel fibers.
3. A significant improvement in the splitting tensile strength was also observed owing to hybrid fiber addition. For instance, 66% increase in splitting tensile strength was recorded for specimens incorporating 1% of hybrid steel fibers. The combination of short and long fibers can better resist the onset of microcracks into macrocracks, thus mitigating the splitting of the matrix and leading to enhanced splitting tensile strength.
4. The tested UHPC specimens achieved superior durability performance indicators. It was observed that the volume of permeable voids was less than 4% for all UHPC mixtures. Further, RCPT coulomb values were less than 100 for the tested UHPC mixtures, indicating exceptional resistance to chloride ions penetrability owing to the negligible porosity of the matrix. This improved durability performance guarantees the long-term performance of UHPC tunnel linings with minimal maintenance and repair costs.
5. Flexural testing of the reduced scale UHPC tunnel lining segments showed that the crack and ultimate loads were highly dependent on the steel fiber dosage. UHPC lining segments without steel fibers attained crack and ultimate loads of 18.10 kN and 18.65 kN, respectively. An increase in the crack and ultimate loads of approximately 41% and 53% was observed, respectively, for UHPC segments incorporating 1% of hybrid steel fibers compared to that of the segment without fibers. Steel fibers restricted the initial development and further propagation of micro-cracks, thus enhancing the load carrying capacity of the lining segments.
6. Greater number of multiple micro-cracks and closely spaced cracks were observed for segments incorporating higher dosage of hybrid fibers due to a greater number of fibers restricting the development of macrocracks.
7. Fiber pull-out behavior and localized failure at a single point was dominant for all the tested UHPC segments incorporating fibers under flexural mid-span load.
8. Hybrid fibers of shorter and longer length in UHPC lining segments improved the stain hardening and strain softening zone owing to their synergistic beneficial effects.
9. Thrust loading tests conducted on the UHPC lining segments without fibers showed cracking and chipping off at the base of the segments. However, no cracks were observed for UHPC lining segments made with hybrid fibers, indicating redistribution of stresses.
10. It can be concluded that UHPC tunnel lining segments made with hybrid steel fibers can be a very competitive design option since it can in many design cases eliminate the conventional steel reinforcing cage and the associated corrosion issues. Moreover, the use of UHPC in tunnel lining segments allows making slender sections, leading to reduced overall weight, easier transport to the job site and simpler installation of the lining segments in-situ.
11. It should be noted that the present study was conducted on reduced scale specimens. Therefore, it is recommended that prototype testing on actual full-scale lining specimens be conducted under various field loading conditions using diverse support systems for gaining greater confidence in the use of UHPC lining segments.
12. Also, lining segments are subjected to various thrust loads depending on the defined jack arrangement of the TBM, which affects the global behavior of the segments. The test set-up used in the present study only replicates a single thrust shoe responsible for stress concentration. More complex actions of thrust load need to be further explored for simulating job site TBM operations in future studies.
13. Although, the initial cost of UHPC tunnel lining segments may be higher in comparison with that of conventional concrete; substantial life cycle cost savings are anticipated in view of the expected lower maintenance and repair, especially in harsh environments.

## Figures and Tables

**Table 1:** Chemical properties of raw materials used for manufacturing UHPC.

Components	Cement (%)	Silica Fume (%)	Quartz Powder (%)	Quartz Sand (%)
CaO	62.51	0.50	0.01	0.01
Al_2_O_3_	3.80	0.15	0.06	0.04
Fe_2_O_3_	3.62	0.09	0.03	0.04
SiO_2_	19.31	95.40	>99	> 99
MgO	2.64	0.37	-	-
SO_3_	3.93	0.20	-	-
K_2_O	1.12	0.52	-	-
Na_2_O	0.15	0.18	-	-
LOI	1.81	1.87	0.11	0.13

**Table 2:** Mixture proportions of UHPC.

Materials	Ratio of Cement Mass
Cement ^1^	1.00
Silica fume	0.25
Quartz powder	0.25
Quartz sand	1.25
Water	0.25
Superplasticizer ^2^	2.50

^1^ Cement = 760 kg/m^3^. ^2^ Superplasticizer by cement mass (%).

**Table 3:** Coefficient of variance of different tests conducted.

Test	Number of Specimens	Coefficient of Variance for Various Fiber Dosages			
		0	1%	2%	3%
Flowability	4	2.10	2.05	2.22	1.85
Compressive strength (75 mm × 150 mm)	5	1.44	1.20	1.52	2.06
Tensile strength (75mm × 150 mm)	5	1.71	1.54	2.11	2.17
VPV (75 mm × 150 mm)	5	1.65	2.23	2.17	1.88
Sorptivity test (75 mm × 150 mm)	5	1.82	2.12	1.56	1.89
RCPT (75 mm × 150 mm)	5	2.11	2.51	1.87	1.60

**Table 4:** Flow and mechanical properties of UHPC mixtures.

Fiber Dosage (%)	Flow Diameter (mm)	Compressive Strength (MPa)	Modulus of Elasticity (GPa)	Tensile Strength (MPa)
0	810	148	39.6	9.50
1	792	157	39.8	15.80
2	773	160	40.2	18.02
3	752	164	40.8	21.52

**Table 5:** Durability properties of UHPC mixtures.

Fiber Dosage (%)	VPV (%)	Sorptivity (kg/m^2^/h^0.5^)	RCPT (Coulombs)
0	3.51	0.0430	70
1	3.27	0.0386	62
2	3.15	0.0348	54
3	3.01	0.0313	46

**Table 6:** Flexural load results of UHPC tunnel lining segments.

Fiber Dosage (%)	Crack Load (kN)	Ultimate Load (kN)
0	18.10	18.65
1	25.55	28.50
2	31.55	36.20
3	39.15	44.80
